# Pathobiology of Cutaneous Manifestations Associated with COVID-19 and Their Management

**DOI:** 10.3390/v14091972

**Published:** 2022-09-06

**Authors:** Waniyah Masood, Shahzaib Ahmad, Noor Ayman Khan, Amaima Shakir, Ghasem Rahmatpour Rokni, Michael H. Gold, Clay J. Cockerell, Robert A. Schwartz, Mohamad Goldust

**Affiliations:** 1 Department of Medicine, Dow Medical College, Dow University of Health Sciences, Karachi 75271, Pakistan; 2 Department of Medicine, Mayo Hospital Lahore, King Edward Medical University Lahore, Lahore 54000, Pakistan; 3 Department of Dermatology, Faculty of Medicine, Mazandaran University of Medical Sciences, Sari 48175866, Iran; 4 Gold Skin Care Center, Nashville, TN 37215, USA; 5 Tennessee Clinical Research Center, Nashville, TN 37215, USA; 6 Departments of Dermatology and Pathology, The University of Texas Southwestern Medical Center, Dallas, TX 75390, USA; 7 Cockerell Dermatopathology, Dallas, TX 75235, USA; 8 Dermatology, Rutgers New Jersey Medical School, Newark, NJ 07103, USA; 9 Department of Dermatology, University Medical Center Mainz, 55131 Mainz, Germany

**Keywords:** skin manifestations of COVID-19, cutaneous manifestations, urticarial eruptions, chilblain-like rash, maculopapular rash, morbilliform rash, erythematous vesicular rash, papulo-vesicular rash, SARS-CoV-2 virus

## Abstract

Severe acute respiratory syndrome coronavirus 2 (SARS-CoV-2) causing coronavirus disease 2019 (COVID-19) has been a rising concern since its declaration as a pandemic by the World Health Organization on 11 March 2020. Recently, its association with multiple underlying organs has been identified that includes cardiac, renal, gastrointestinal, nervous systems, and cutaneous manifestations. Cutaneous COVID-19 findings have been supposedly classified into the following categories: vesicular (varicella-like), papulo-vesiculsar, chilblains-like (“COVID toes”) maculopapular, and urticarial morphologies. In this review, we aim to focus on the proposed pathophysiology behind the various dermatological manifestations associated with COVID-19 and their associated management. We also included prevalence and clinical features of the different COVID-19-related skin lesions in our review. A comprehensive narrative review of the literature was performed in PubMed databases. Data from case reports, observational studies, case series, and reviews till June 2022 were all screened and included in the review.

## 1. Introduction

More than five million deaths have been a consequence of the emerging lethal coronavirus (COVID-19) pandemic [1]. The transmissible plague caused by SARS-CoV-2, an enveloped RNA virus, has been detrimental to the world population since its commencement in Wuhan, China in 2019 [2]. Omicron, a novel mutated variant of the SARS-CoV-2 virus, with an expected increased rate of transmissibility and partial vaccine resistance is also an ongoing concern [3]. Major clinical manifestations of coronavirus disease include respiratory and pulmonary illnesses (fever, cough, anorexia, anosmia, and dyspnea) are a few of the major signs and symptoms. Other than the above stated, there are also extrapulmonary complications comprised of multiple organ systems such as renal, gastrointestinal, hepatic, cardiac, nervous, hematological, and cutaneous manifestations [2].

Ever since a study by Recalcati reported cutaneous involvement in 20% of patients with confirmed SARS-CoV-2 virus infection in a hospital in Italy [4], dermatologic manifestations have become a center of notoriety for medical researchers. According to a registry analysis by MacMahon et al., a total of 331 patients infected with the virus reported cutaneous manifestations from 41 countries in 5 months [5]. The increase in dermatologic manifestations amongst COVID-19 patients in the past few months has become an enigma. Multiple researchers have identified some major similar patterns of cutaneous involvement associated with the SARS-CoV-2 virus that includes: urticarial rash/hive-like rash, erythematous/maculopapular-like rash, papulo-vesicular exanthema, and chilblain-like rash [6,7]. A study reported the prevalence of different cutaneous rashes and found morbilliform rash in 22% of individuals, pernio-like acral lesions in 18%, urticaria in 16%, macular erythema in 13%, vesicular eruption in 11%, and papulo-squamous eruption in 10% of cases [7]. A similar recent study by Jamshidi et al. evaluated that the mean overall prevalence of cutaneous involvement in COVID-19 patients was 6%, with maculopapular lesions having the highest prevalence, that is in around 40% of cases, followed by chilblain-like rash with 20% [8]. Associated signs and symptoms of cutaneous involvement ranges from mild to severe, with fewer cases reported with pruritus. The time duration of different rashes varies accordingly, urticarial and maculopapular have a shorter duration while chilblain-like rash reported staying for a longer time duration [7]. Females showed a slighter higher prevalence of cutaneous involvement as compared to males [8].

Albeit having significant cases associating cutaneous involvement in COVID-19 patients, the underlying cause and pathophysiology have still not been identified from the data. It has been expected that the interaction of the SARS-CoV-2 virus transmembrane spike glycoprotein with angiotensin-converting enzyme 2 (ACE2) could be a potential cause of cutaneous involvement when considering the fact that various skin cells like basal epidermal cells, sebaceous glands, and keratinocytes express ACE2 receptors [9]. Thus, in this review we aim to summarize the hypothetical phenomena behind the pathophysiology of different cutaneous manifestations associated with COVID-19 and any treatment modalities available for each condition. 

## 2. Materials and Methods

A comprehensive narrative review of the literature was performed of PubMed databases. Data from case reports, observational studies, case series, and reviews till June 2022 were all screened and included in the review. A combination of coronavirus search terms (COVID-19, SARS-CoV-2, Coronavirus) and dermatological search terms (skin manifestations, cutaneous manifestations, urticarial eruptions, chilblain-like rash, maculopapular rash, morbilliform rash, erythematous vesicular rash, papulo-vesicular rash) were used. The references of all articles were then screened, and relevant articles were added.

## 3. Results

Using the keywords skin manifestations and COVID-19, there were 751 articles found on PubMed, which were further screened and relevant data from publications in English were included. Articles regarding specific kinds of skin manifestations being discovered in either suspected/positive COVID-19 patients were also searched for in PubMed. Urticarial eruptions and COVID-19 (*n* = 101), chilblain-like rash and COVID-19 (*n* = 25), maculopapular rash and COVID-19 (*n* = 86), morbilliform rash and COVID-19 (*n* = 28), erythematous vesicular rash and COVID-19 (*n* = 8), papulo-vesicular rash and COVID-19 (*n* = 14), and cutaneous manifestations and SARS-CoV-2 (*n* = 337) was also used in PubMed to widen the search for any publication in relation to our topic.

### 3.1. Urticarial Eruptions and COVID-19

#### 3.1.1. Prevalence and Associated Clinical Features

Urticarial rashes are characterized by the development of red, itchy, and elevated cutaneous wheals of varies sizes. Urticaria is a cutaneous disorder comprised of either wheal (hives), angioedema, or both consecutively [10]. Multiple researchers have evaluated the association of urticaria rash with several underlying diseases, such as autoimmune thyroid diseases, diabetes mellitus, systematic lupus erythematosus, coeliac disease, rheumatoid arthritis, and asthma [11]. Urticaria may also be a paraneoplastic sign of malignancy. Recently, urticarial cases in patients tested positive for the SARS-CoV-2 virus showed a possible correlation between the two diseases. Since the start of 2020, multiple case reports have highlighted urticaria in COVID-19 patients. A study by Galván Casas et al. reported a total of 73 patients with COVID-19 with urticaria [12]. Likewise, a study by Freeman et al. reported 27 patients with confirmed COVID-19 and urticarial [13]. A cohort study by Recalcati showed that urticarial eruptions account for 16.7% of cutaneous manifestations in COVID-19 patients [4].

**Figure 1:**
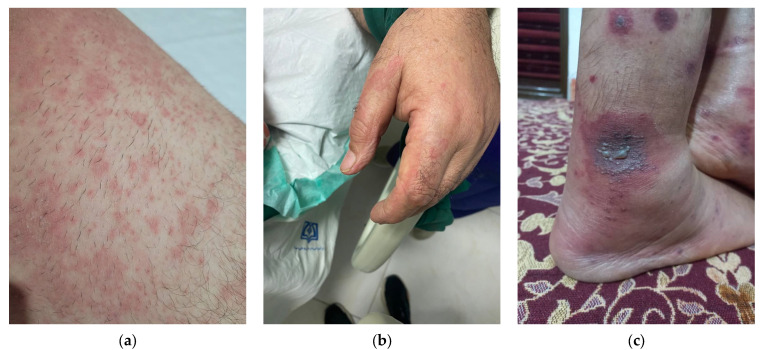
Cutaneous manifestations of COVID-19. (**a**) Papulo-squamous lesions (**b**) wheal (**c**) petechia purpura and vasculitis.

Similarly, a study by Marzano et al. classified cutaneous manifestations in different groups identified from 375 case reports and found that urticarial eruptions accounted for 19% of total cases [7]. Several researchers have also accentuated that those urticarial eruptions are mostly reported with the onset of other symptoms of COVID-19 such as cough, fever, chills dyspnea, fatigue, and other non-cutaneous COVID-19 symptoms, angioedema may or may not be present. Urticarial rash has also been recorded to precede other common symptoms of COVID-19 (cough, dyspnea, fever) [14]. The rash is self-limiting, and disappears as early as 24 h with a better prognosis rate of 98% [14,15,16]. Some common sites for the rash appear to be the trunk, hands, and feet, with a mean age of 38 years and female predominance [14] (Figure 1).

#### 3.1.2. Pathophysiology

##### Mast-Cell-Mediated Response

**Figure 2:**
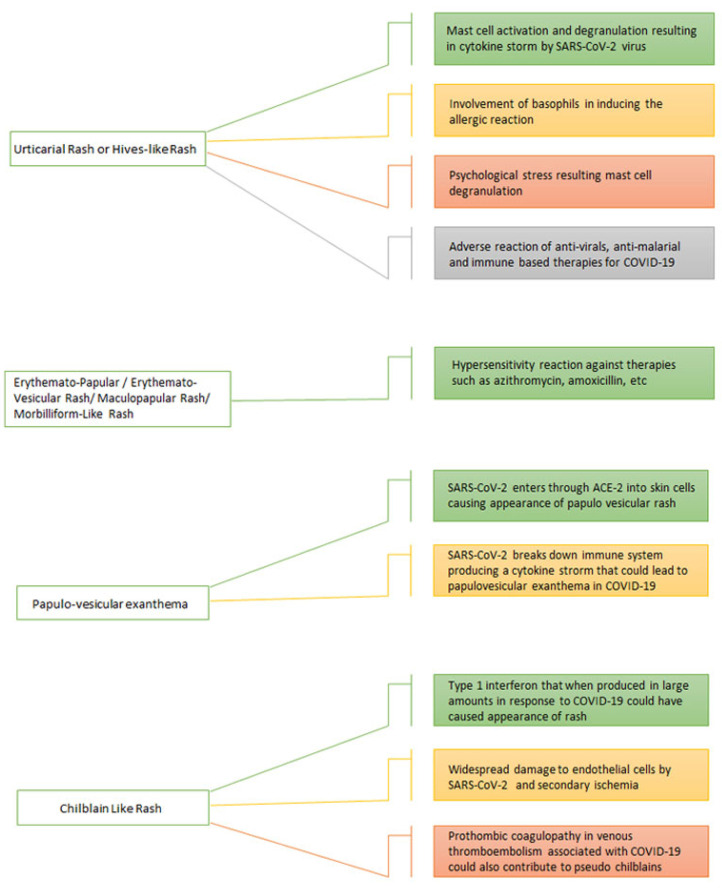
Pathogenesis behind cutaneous manifestations of COVID-19.

Mast cells are considered as principal mediators in the pathogenesis of urticarial rash [17]. Bacterial infection induces the disease by activating toll-like receptors (TLR4, 7 and 9), high-affinity IgE receptor (FcεR1), and complement receptors to release pro-inflammatory mediators, cytokines, histamine, and other mediators resulting in leukocyte infiltration, sensory nerve activation, vasodilatation, and intradermal edema [17,18]. Although the exact pathogenesis correlating urticarial rash and SARS-CoV-2 virus is unknown, nonetheless there are a few hypothetical explanations for the mechanism behind it (Figure 2).

Molecular hypothesis reflects the co-relation of IL-6 that is associated with SARS-CoV-2 infection with the pathogenesis of urticaria. As it has already been known that mast cell activation resulting in cytokine storm is the main cause behind the pathogenesis of inflammatory response and allergic reactions in viral or bacterial infections. Elevated levels of cytokines in COVID-19 such as tumor necrosis factor-alpha (TNF-α), interleukin-6 (IL-6), IL-1β, granulocyte-macrophage colony-stimulating factor (GM-CSF), and chemokine (C-C-motif) ligand 2 (CCL2) that are primarily released by mast cells proved to be a substantial indication involving mast cell activation in COVID-19 patients [18]. Moreover, stress related to COVID-19 infection further potentiates pro-inflammatory cytokines that are involved in the pathogenesis of urticaria.

##### Eosinophils Infiltration

Besides this, Dastoli et al. proposed the concept of eosinophilia associated with urticaria in COVID-19 patients [16]. However, a study by Algaadi et al. observed eosinophilia in 11 COVID-19 patients, out of which only 18% of patients developed systematic eosinophilia, thus indicating an insignificant correlation between them [19].

##### Basophils Infiltration

Basophils could also be involved in the pathogenesis of the development of urticaria in COVID-19 patients. Basopenia has been observed in the setting of COVID-19 patients similar to chronic urticaria, showing a potential correlation between them [19].

##### Stress-Induced Response

Notably, chronic low-grade inflammation has repeatedly been associated with the majority of neuropsychiatric disorders, suggesting a persistent low level inflammation all across the body. Around 95% of COVID-19 patients experience emotional and psychological stress during the diseases [20]. As stress is a potent factor in instigating mast cell degranulation through neuropeptides and glial cells, and inducing a cytokine storm, thus it could be a possible reason for inducing urticarial rashes in the COVID-19 patients [21]. This suggests a psychoimmunological model of disease pathology.

##### Drug Hypersensitivity

Besides this, certain anti-COVID-19 medications such as, lopinavir/ritonavir, nitazoxanide, corticosteroids, baricitinib IVIG treatments, and checkpoint inhibitors are also suspected to induce side-effects involving urticarial rashes on the body [22]. Generalized pustular figurate erythema (GPFE) is a distinctive severe cutaneous drug reaction with widespread urticarial plaques topped with non-follicular pustules that sometimes evolve targetoid plaques in annular and arcuate patterns. It is a medication eruption seen with hydroxychloroquine. However, hydroxychloroquine was first used in the management of SARS-CoV-2 but recent guidelines favor corticosteroids and anti-viral agents [23,24]. Thus, drug reactivity could also be a potential reason behind the pathogenesis of disease.

#### 3.1.3. Management

Managing urticaria amongst COVID-19 patients could be inconvenient due to the limited therapeutic options available [19]. Some therapies for COVID-19 management including hydroxychloroquine, azithromycine, and oseltamivir are reported to trigger an inflammatory response and thus could be a potential cause of urticaria [25]. Possible therapeutic options to treat the condition includes nonsedating antihistamine and low dose corticosteroids [26] (Table 1).

### 3.2. Erythemato-Papular or Erythematous-Vesicular Rash or Maculopapular Rash or Morbilliform-like Rash

#### 3.2.1. Prevalence and Associated Clinical Features

Maculopapular rash has been a key feature of many viral infections being associated with measles, echovirus, rubella, HIV, and roseola. Besides being common in the aforementioned viral diseases, it has also been reported with COVID-19. Bacterial conditions involving maculopapular rash are scarlet fever, erysipelas, erythema marginatum, rocky mountain spotted fever, and lyme disease. Literature is replete with similar presentations in COVID-19 cases. An abrupt eruption of erythematouspapular lesions involving the trunk and lower limbs in a peritoneal dialysis patient was described by Alvarez et al. in 2021. The existence of moderate chronic and superficial purpuric dermatitis in association with COVID-19 was revealed by a punch biopsy of the abdomen [27]. In some cases, the rash may be maculopapular but, later on, it may progress to other cutaneous manifestations, as reported by Jimenez-Cauhe et al. in 2021. They described three cases in which erythematous papules appeared on the upper trunk and progressed to erythematous-violaceous patches with a dusky core and a pseudovesicle in the middle. Two of the patients had typical target lesions. Lesions began to coalesce in the back, then spread to the face and limbs within a week, with palms and soles remaining unaffected. The oral cavity of three patients was examined, revealing palatal macules and petechiae [28]. A similar study by Caputo et al. described a COVID-19 positive woman with a morbilliform rash whose skin biopsy revealed certain cytopathic epidermal alterations at the tissue level that revealed histopathological findings that are characteristic of SARS-CoV-2 infection. The presence of viral particles in keratinocytes, as well as the immunohistochemically positivity of endothelial cells and eccrine glands using anti-SARS-CoV-2 Spike S1 antibodies, suggested that the lesions were caused by SARS-CoV-2 infection [29].

The association of maculopapular rash with pruritis and itching is well pronounced. In Spain, 175 COVID-19 patients had a maculopapular rash, most commonly in the adult population. It commonly appeared alongside other classic COVID-19 symptoms with more than (56%) half reporting itching. It lasted approximately 9 days and was linked to more severe coronavirus infection [12]. A 67-year-old Italian woman suffering from alcohol dependence with COVID-19 presented with an erythematous pruritic rash with palmoplantar and facial skin-sparing, as well as mucosal sparing. A skin biopsy revealed a modest superficial perivascular lymphocytic infiltration in the upper and mid dermis, as well as dilated arteries [30]. A 58-year-old Hispanic male with no fever developed a morbilliform purpuric rash that lasted 6 days after the appearance of the lesions. He had a history of azithromycin and benzonatate drug intake. Legs, thighs, forearms, arms, shoulders, and trunk were all covered in erythematous sores. They coalesced into larger, confluent erythematous spots on the trunk. The face, hands, and feet were spared, and there were no reports of intraoral complaints [31] (Figure 1).

#### 3.2.2. Pathophysiology

##### Drug Hypersensitivity

The pathophysiology of maculopapular lesions varies greatly in COVID-19 cases. The histopathological findings may reveal Grover-like characteristics, lymphocytic vasculitis, and microthrombi in the elderly [32]. Minor spongiosis, basal cell vacuolation, and mild perivascular lymphocytic infiltrate were found in diffuse fixed erythematous blanching asymptomatic maculopapular lesions. Drug reactions may also manifest as a maculopapular rash. COVID-19 can cause a standard-appearing viral exanthem on its own or in a patient who has been sensitized to azithromycin or benzonatate, much like an Epstein–Barr virus infection can create a cutaneous eruption on its own or in a patient who has been sensitized to amoxicillin [33] (Figure 2).

Exanthematous maculopapular eruption, also known as morbilliform (measles-like) rash, could be the result of a medication hypersensitivity reaction. It is marked by a widespread, and sometimes generalized, symmetric eruption of erythematous macules and/or papules that appear one to two weeks after starting therapy with the causative substance, or as early as 6 to 12 h, and up to three days in previously sensitized individuals. In the current scenario, the drug therapy being employed to curb COVID-19 may be contributing to the eruption of maculopapular rash in COVID-19 cases.

#### 3.2.3. Management

Topical steroids helped to clear up the exanthema and maculopapular rash. To treat dermatitis, a 0.1 percent cream of triamcinolone was recommended resulting in relief from the symptoms [31] (Table 1). However, oral steroids have also been prescribed in maculopapular lesions associated with SARS-CoV-2 infection and they have shown promising results. Alvarez et al. reported that a patient who was given 30 mg of prednisone, with a weekly tapering dose of 10 mg, recovered completely after ten days of treatment [27].

### 3.3. Papulo-Vesicular Exanthema

#### 3.3.1. Prevalence and Associated Clinical Features

Although skin presentations were rare during the onset of the spread of the illness, a study conducted in January 2020 soon reported two patients with rashes. The data used in the Chinese research study consisted of 1099 confirmed cases of COVID-19 laboratory samples [34]. Among many of its manifestations, papulo-vesicular exanthema or varicella-like vesicular lesions are commonly seen [35].

The first clinical report of SARS-CoV-2 associated with cutaneous involvement came from Italy. The study involved a total of 88 patients who were either directly or indirectly observed. Results illustrate that 18 of these patients showed cutaneous involvement, out of which 1 patient had developed varicella-like vesicles [4]. Following these initial cases, multiple reports have been published showcasing the cutaneous manifestations of COVID-19. A multicenter report from Italy portrays papulo-vesicular exanthem as a specific dermatologic manifestation of COVID-19. The study also established that the vesicular exanthem associated with SARS-CoV-2 was unlike true varicella as it had typical features of trunk involvement, scattered distribution of rash, and mild or absent itching [7]. Furthermore, a nationwide study conducted in Spain presented 34 cases of papulo-vesicular exanthem, which comprises 9% of the total 375 cases observed [12]. The study also described the vesicular lesions to be monomorphic instead of the usual polymorphic varicella lesions, making them specific to COVID-19 and distinguishing them from other viral exanthems [12]. With respect to French observational studies, 7 cases of inflammatory lesions were noted out of a total of 14 cases. Of these 7 cases of inflammatory lesions, 2 embodied vesicular lesions [36]. In addition to the European and Chinese research studies, a study from Turkey examined 210 hospitalized cases of cutaneous manifestations, 5.8% of whose patients displayed symptoms of vesicular rash. Moreover, 53 patients from a set of 678 patients had shown symptoms of new dermatological conditions from which 4% consisted of vesicular lesions [37].

#### 3.3.2. Pathophysiology

In the spring of 2020, Madrid’s Ramón y Cajal University Hospital further split the papulo-vesicular rash into two categories: diffused and localized. In circumstances where the exanthem sample is diffuse, it would be widespread and display a polymorphic rash, whereas the localized rash would be monomorphic and tended to predominantly be on the trunk [38]. In this study, out of 24 samples recorded, a rash was seen after the development of COVID-19 symptoms in 19 samples. The vesicular rash was found to approximately last for 10 days [39]. The Spanish institute also conducted a histopathological examination of two lesions which lead to the discovery of vesicles with acantholysis and ballooned keratinocytes [38]. These papulo-vesicular manifestations would lead to differentials of varicella-vesicles also commonly known as chickenpox among the general public. Furthermore, differentials could also be herpes-associated vesicles and COVID-19 vesicles, with overlapping clinical symptoms [40].

As discussed earlier, due to the presence of ACE2 in skin cells, there is a possibility of the enzyme being the cause of cutaneous manifestations associated with COVID-19 [39] (Figure 1). One of these studies hypothesized that cytokine storms cause cutaneous manifestations due to SARS-CoV-2’s ability to break the human innate immune system while promoting a cytokine response. Researchers conclude that this phenomenon could eventually lead to the cutaneous manifestation caused by its direct viral involvement and its cytopathic effect [41].

#### 3.3.3. Management

While there is no current treatment available to patients suffering from papulo-vesicular exanthem, the conservative method or the wait-and-see method are the preferred ways of dealing with its symptoms (Table 1). Healthcare professionals often ascribe to these methods as the symptoms are not considered to be severe, and usually tend to resolve by themselves [6]. Finally, vesicular lesions have been described as a specific form of cutaneous manifestations with regards to the SARS-CoV-2 virus.

### 3.4. Chilblain-like Rash

#### 3.4.1. Prevalence and Associated Clinical Features

According to a case series in Spain that classified skin manifestations in confirmed or suspected COVID-19 patients into five kinds, the chilblain-like rash was one of them [42]. First discovered in a COVID-19 confirmed patient, a thirteen-year-old boy with acral-like chilblain lesions on 29 March 2020, in Italy [15]. Later on, similar acral lesions on the feet of suspected or confirmed COVID-19 patients were circulated via social media among dermatologists in Italy and France [43].

This cutaneous feature was then found to be prevalent in Europe, the United States, and the Middle East [15,44]. Further proved in a study where out of 505 patients from eight different countries including the United States, France, and Italy; pseudo-chilblain was found to be present in 318 patients (63%) [45].

It was seen to be more common in Caucasians than any other ethnic groups [6], and the age demographic showed pseudo chilblains were reported to occur frequently in children and younger adults who were either asymptomatic or displaying mild symptoms [46]. Except for chilblain-like rash, there was no other dermatologic manifestation seen in asymptomatic COVID-19 patients. This was further confirmed in the same case studies stated above; where in 55% of the patients, pseudo chilblains were the only symptom found [45]. Radiological abnormalities like lung infiltrates were also less associated with chilblain-like rash than any other dermatological lesions. Moreover, there was also a decreased hospitalization and ICU admission rate seen in patients having a chilblain-like rash [47].

Due to the lack of correlation of the rash with exposure to cold weather but having a similar appearance to chilblains, it was named pseudo-chilblains [15]. Termed also as “COVID toes”, the lesions were located on acral surfaces, i.e., the hands and feet, specifically the fingers and toes [42]. The pattern for pseudo chilblains varied from erythematous to purple, purpuric macules, papules, or vesicles [48]. The lesions were mostly asymmetrical and presented with pain and itching [42].

**Table 1:** Summary of cutaneous manifestations of COVID-19.

Type of Rash Associated with COVID-19	Prevalence (%)	Mean Age (Years)	Gender Predominance (Male/Female)	Clinical Features	Associated Symptoms	Healing Duration	Treatment	Survival Rate (%)
Urticarial Rash [4,14]	15–20%	38	Female	Red itchy elevated rash on trunk and limbs	Cough Dyspnea Fever Chills Angioedema	24 h	Low dose corticosteriodAnti-histamines	98%
Erythemato-Papular/Erythematous-Vesicular Rash/Maculopapular Rash/Morbilliform-like Rash [28]	45%	60	Male	Erythematous pruritic rash having both flat and raised areas on trunk and limbs	cough fever pneumonia	1 week	Oral and tropical steroids Triamcinolone	100%
Papulo-vesicular exanthema [7]	4–9%	40–60	Female	Widespread, varied sized polymorphic papules or vesicles Itchy, monomorphic vesicles on trunk	None	8 days	Conservative/Observational	98%
Chilblain-like rash [42]	16–30%	13–20	Both	Red to purple, itchy, painful rash on toes and fingers	None	1–2 weeks	Conservative/Observational Tropical corticosteroids	98%

In a case study, 15 patients were divided into two groups, where the group with an active phase of the disease displayed through dermoscopy red dots that were congested, enlarged vessels surrounding white rosettes and white streaks on a pinkish-reddish background. Whereas, those patients in the remitting phase showed fewer red dots, white streaks, and blurred rosettes [49]. Compared to the other skin manifestations, chilblain-like lesions were found later in the course of COVID-19 disease following a latency period post other symptoms and lasted for an average of one or two weeks [9,50]. On resolution, there could be post-inflammatory hyperpigmentation [42].

Histologically, regenerative changes in the epidermal region, perivascular lymphocytic dermal infiltrate, and vacuolar degeneration in focal areas of the basal layer were seen in samples of pernio-like rash associated with COVID-19 [51]. A study also showed that chilblain-like rash displayed histological features of necrotic epidermal keratinocytes, papillary dermis edema, and inflamed perieccrine sweat gland with predominant dermal infiltrating CD3+/CD4+ T cells that have been similarly found in autoimmune-related chilblains and idiopathic chilblains [52].

#### 3.4.2. Pathophysiology

##### Type 1 Interferon Response

One of the hypotheses for pernio-like lesions was a link with type 1 interferon that when produced in large amounts in response to COVID-19 could have caused the appearance of the rash [45]. 

##### Endothelial Cell Damage and Ischemia

Another potential pathophysiological cause behind these skin manifestations could be widespread damage to endothelial cells by SARS-CoV-2 and secondary ischemia. This hypothesis was discovered in a research study where skin biopsies of seven pediatric patients were analyzed by immunohistochemistry and electron microscopy [53].

##### Prothrombotic Coagulopathy

A less common contributing factor could be prothrombotic coagulopathy seen in complex cases of venous thromboembolism in COVID-19 [45] (Figure 1).

Endothelialitis, vascular microthrombi in superficial dermal capillaries and eosinophilic fibrin deposition in the wall of the dermal venules found in cases of chilblain-like rash further strengthens these hypotheses. The finding of vascular deposits of IgM, IgA, or C3 by direct immunofluorescence examination on 14 of the 17 skin biopsies performed on patients with chilblain-like lesions in a study is evidence of vascular injury being involved in the pathogenesis of this cutaneous manifestation [52].

#### 3.4.3. Management

A proper history and physical examination of a patient presenting with acral lesions should be done to exclude any other differential diagnosis for chilblains, especially, exposure to cold which is uncommon in COVID-19-associated chilblains [42]. Due to patients presenting with chilblain-like skin lesions being asymptomatic or mildly symptomatic, it is recommended for them to be tested for SARS-CoV-2 to be able to decide on a therapeutic management course [50]. Most of the pseudo chilblains cases reported resolved spontaneously without any need for treatment [53]. However, according to a pediatric case study, the addition of a topical corticosteroid may decrease the duration of the rash and help control symptoms, for example, itching or edema [50] (Table 1).

### 3.5. Other Cutaneous Manifestations

A study reported a patient with a mildly irritating erythematous rash that first affected the patient’s extremities (hands, feet, forearm, legs, and back surface of the ears) in the form of folliculo-centric papules accompanied with pruritus. These papules progressed across her body, sparing only her face, scalp, and abdomen. The patient was prescribed cetirizine 10 mg once a day to cure the skin rash. With the use of an oral H1-antihistamine, the itching rash improved and was relieved after ten days. However, keratosis-pilaris-like tiny papules on her arms persisted, giving her skin a stippled appearance like gooseflesh. These lesions were described as felt by the patient, although they were not apparent during the distant evaluation. Over time, these little, perceptible papules faded [54].

A scarlet fever-like eruption with subsequent furfuraceous desquamation has also been reported. Two such cases have been described by Birlutiu et al., 2020. Fever preceded the rash in all cases, and the rash was generalized [55].

The SARS-CoV-2 infection has also been linked to androgenetic alopecia. In a clinical investigation of 41 Caucasian males with mild to severe bilateral COVID-19 pneumonia, these patients suffered from androgenetic alopecia. They had a mean age of 58 years, 29 (71%) had moderate androgenetic alopecia and 12 (29%) had severe alopecia based on the Hamilton Norwood Scale [56].

Pityriasis rosea-like eruption may also occur with COVID-19. Veraldi et al. described two cases of pityriasis rosea-like eruptions in patients infected with COVID-19. The rash had been present for a few days and was accompanied by pruritus, mild headache, and arthralgia. Several erythematous squamous papules and plaques were discovered on the upper limbs and trunk during a dermatological examination [57].

SARS-CoV-2 infection requires the androgen-regulated TMPRSS2 protease, which is a cellular coreceptor. This enzyme prepares the viral spike protein. Because androgens decrease the immune system, another link is androgen-driven immunological regulation. The 3-hydroxysteroid dehydrogenase-1 gene, which is involved in the translation of dehydroepiandrosterone into active and more potent androgens, is encoded by the adrenal permissive phenotype of the HSD3B1 gene. The biological plausibility of SARS-CoV-2 infection explains its link to the pathophysiology of androgenetic alopecia [56] (Figure 1).

Pityriasis-rosea-like eruption associated with COVID-19 can be managed by peripheral H1 receptor blocker. Cetirizine was prescribed at a dose of 10 mg per day for three weeks. The skin lesions and other symptoms disappeared with the therapy [57] (Table 1). Similar pityriasis-rosea-like lesions have also been reported with a few cases who underwent vaccination against SARS-CoV-2 [58,59].

### 3.6. Cutaneous Manifestations in Viral Variants of COVID-19

SARS-CoV-2, the virus that causes COVID-19 is known to undergo mutations and change over time to form different variants. According to WHO, there are five known variants of concern namely, Alpha, Beta, Gamma, Delta, and the latest Omicron. With these variants, the virus is known to undergo changes in transmissibility, virulence, clinical presentation, and the ability to escape public health measures [60]. With the newer variants there also is variability in the presentation of cutaneous manifestations. According to a study conducted in the United Kingdom, 7430 (17.6%) participants infected with the Delta variant had dermatological symptoms, while the number of participants with the Omicron variant having dermatological findings were 8632 (11.4%) showing a decrease in the frequency of dermatological symptoms in the newer Omicron variant [61]. The study also reveals the timing after which the cutaneous findings presented on average, which was 6 and 5 days for the Delta and Omicron variants, respectively [61]. Likewise, an observational study based on the ZOE COVID-19 App compares symptom prevalence amongst the new variants and reveals a significantly lower frequency of cutaneous manifestations in both Delta and Omicron variants [62]. The skin manifestations seen in the Omicron variant include skin discoloration followed by a decrease in oxygen delivery, a rash resembling hives that frequently affects the palms and soles, and miliaria rubra, also known as prickly heat rash that affects the entire body [52]. A few other symptoms include eczema on the neck and chest, swollen lips, and inflamed toes [52].

A retrospective cohort study comparing outcomes of the new variants shows a significant decrease in both emergency department visits and hospitalization amongst the population infected with the Omicron variant and hence resulting in an overall decrease in cutaneous findings [63].

### 3.7. Cutaneous Findings in Long-Standing COVID-19 Patients

The NICE guidelines provide two definitions of post-acute COVID-19, i.e., ongoing symptomatic COVID-19 for people who still have symptoms between 4 and 12 weeks after the start of the acute phase of disease; and post-COVID-19 syndrome for people who have persistent symptoms for more than 12 weeks [64]. These prolonged symptoms include fatigue, loss of sense of smell and taste, difficulty in concentration, hair loss and skin lesions. A cross-sectional survey held in Germany showed that 26 out of 588 non hospitalized patients and 15 out of 127 hospitalized patients presented with skin lesions that persisted for more than 12 weeks [65]. While a cohort study carried out in China reported skin rash as a symptom occurring 6 months after onset of COVID-19 infection in only 47 out of 1655 patients, i.e., 3% [66].

According to the International Dermatology Registry definition of long-haulers as patients presenting with skin manifestations for more than 60 days, pernio skin lesions and livedo reticularis lasted for the longest duration among the cutaneous manifestations associated with COVID-19 [5]. While urticarial skin lesions and morbilliform rash had the shortest duration with each persisting for a median time period of 4 and 7 days, respectively [67].

### 3.8. Skin Lesions in Vaccinated Individual

Vaccinated individuals owing to the decrease in viral load if later become infected with SARS-CoV-2 experienced milder symptoms. Therefore, patients who had been vaccinated but later on become infected with COVID-19 developed only mild cutaneous findings for example, urticarial and vesicular eruptions [68]. There appears to be limited data compiled on dermatological manifestations in individuals with COVID-19 infection who had previously been vaccinated. Thus, it is an area of study that could be further researched as vaccinations become more common.

## 4. Conclusions

Following Recalcati’s discovery of COVID-19’s cutaneous manifestations, various investigations have demonstrated the variety of the inflammatory and vascular skin lesions caused by COVID-19. The prevalence of cutaneous symptoms in COVID-19 patients ranges from 5% to 20%. Some frequently observed are vesicular (varicella-like), papulo-vesiculsar, chilblains-like (“COVID toes”) maculopapular, and urticarial eruptions. The possible underlying mechanism of rash includes (i) entrance of the virus through ACE-2 receptors in the skin cells, (ii) extensive endothelial cell injury resulting in subsequent necrosis, (iii) mast cell degranulation and involvement of inflammatory cells, and (iv) hypersensitivity reaction to drugs. Even though the precise pathobiology underlying the reported manifestations is not yet understood, knowing the mechanism underlying the COVID-19-related dermatoses could assist in differentiating it from the other differential diagnoses, rule out any drug-related reasons, help in giving management to curb any associated symptoms from itching to angioedema, and may even play a role in identifying COVID-19 in patients. While serious cutaneous involvements are extremely uncommon, low-dose corticosteroids and anti-histamines are some of the treatments recommended to control cutaneous involvement of COVID-19.

## 5. Strength and Limitations

The prevalence, clinical consequences, management, and potential processes behind the cutaneous symptoms of COVID-19 have all been highlighted and summarized in our review as a whole. The lack of critical evaluation and quality assessment of the reported papers listed is one of the study’s weaknesses. To more accurately assess the pathobiology underlying each of the cutaneous symptoms, more clinical information would be needed. The thorough COVID-19 symptomatology research in the mucosa, hair, and nails was not taken into consideration during the analysis. There has been no anticipated registration for the review paper.

## Data Availability

Data sharing not applicable.
